# How Mean is the Mean?

**DOI:** 10.3389/fpsyg.2013.00451

**Published:** 2013-07-23

**Authors:** Craig P. Speelman, Marek McGann

**Affiliations:** ^1^School of Psychology and Social Science, Edith Cowan University, JoondalupWA, Australia; ^2^Department of Psychology, Mary Immaculate College, University of LimerickLimerick, Republic of Ireland

**Keywords:** mean, average, variability, noise, distributional analyses, cognition

## Abstract

In this paper we voice concerns about the uncritical manner in which the mean is often used as a summary statistic in psychological research. We identify a number of implicit assumptions underlying the use of the mean and argue that the fragility of these assumptions should be more carefully considered. We examine some of the ways in which the potential violation of these assumptions can lead us into significant theoretical and methodological error. Illustrations of alternative models of research already extant within Psychology are used to explore methods of research less mean-dependent and suggest that a critical assessment of the assumptions underlying its use in research play a more explicit role in the process of study design and review.

## INTRODUCTION

Psychology prides itself on its empirical basis. All undergraduate psychology courses focus on training students in methods for collecting and analyzing data about human behavior. To this end, a common introductory lesson in psychology involves measuring a group of humans on some variable and calculating the mean of the values. This mean value is then discussed as representing the average performance of the group, as if this value provides a representative substitute for the group’s data. It is our view that rationalizing a set of data into one value is a theoretically loaded practice that can be misleading and possibly erroneous. While the mathematical tool itself is theory neutral, its use within the community of scientific practice within psychology is not. In this paper we argue that the mean, used without care, can cause illusions of stability and reliability in behavioral data, which in turn leads to inappropriate conclusions regarding the underlying nature of the psychological system. Our principal intent in what follows is to make the assumptions inherent in typical scientific practices more explicit, to expose them for critique. Our examination of the mean is therefore less a statistical one than a theoretical one. Having articulated these concerns we explore a number of related alternative theoretical perspectives that are less reliant on those explicated assumptions. We argue that, at the very least, we should take more care in our use of the mean in analyzing data. Better yet would be the adoption of methods and theoretical frameworks that cope better with the complexity and variability of behavior and cognition.

## THE MEAN AND ITS USAGE

The most common form of the mean that is used in psychology is the arithmetic mean. This represents the sum of all of the values in a set divided by the number of values in the set. Although other forms of the mean are used in psychology (e.g., geometric, harmonic), our arguments are mainly confined here to the arithmetic mean. Our concerns, though, are mainly with the use of measures of central tendency, so our arguments apply in the general sense to all forms of the mean.

Textbooks used in introductory psychology courses on statistics and research methods typically refer to the mean as a measure of central tendency and often contrast it to other measures of central tendency such as the mode and the median. All such measures are used in situations where data sets contain some variation (i.e., not every score has the same value). The aim in calculating one of these measures, then, is to generate a value that sits somewhere in the middle of the distribution of scores. On the relatively rare occasions that the particular importance of measures of central tendency is mentioned it is as an indicator of the typical or most likely scores within the distribution (e.g., Haslam and McGarty, 2003, p. 135; Dancey and Reidy, 2008, p. 44; Howitt and Cramer, 2011, p. 25). These “typical” scores are “summary” or “descriptive” statistics, providing at least some insight into the basic characteristics of the distribution in question.

**FIGURE 1:**
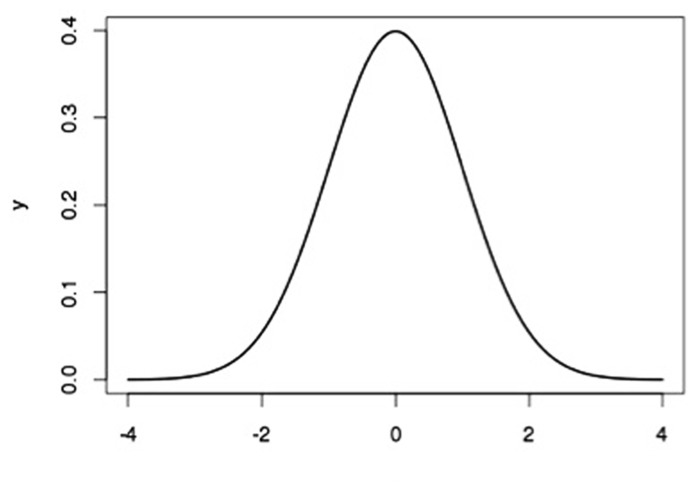
**The Gaussian, or “normal,” distribution**. *X* represents the measured variable, and Y represents the probability of occurrence of particular values of *X*.

However, while all of the data in a distribution are involved in calculation of the arithmetic mean, it remains a matter of judicious use as to how well the mean *represents *those data. Summaries are vital to good communication but used too frequently and uncritically they provide an impression of reliability or consistency that distorts the normal state of affairs. It is this sometimes careless overuse of the mean and its too frequent use without other statistics as a summary of distributions with which we are concerned here. In particular, the over-reliance on the mean (despite the fact that we all, of course, know better) expresses a way of thinking about distributions and variability that we believe poses potentially grave problems for our science. Introductory textbooks typically indicate that most measures of anything related to humans, and indeed any biological system, produce a distribution of scores. These distributions are commonly normal in shape (although see Micceri, 1989). That is, they have a shape that corresponds to the Gaussian distribution, which has a symmetrical shape, with most scores clustered around the middle of the distribution, fewer scores at the tails, and a smooth transition from the middle to the tails (see **Figure 1**). The mean, therefore, sits perfectly in the middle of the normal distribution (as does the median and the mode).

The Gaussian distribution is also referred to as the “normal law of error” (Boring, 1920), suggesting that scores on either side of the mean represent some error of measurement. Adolph Quetelet, the first to apply this law to social and biological data, suggested that the mean of a distribution of human measurements, such as of a set of heights, represented nature’s ideal value and that values on either side of the mean were deviations from nature’s ideal (Howell, 2002)^1^

Relying on the mean of a set of scores to represent the set appears to carry with it the assumption that the variation in values observed around the mean is somehow erroneous. Whether it be that our methods of measurement are faulty, or that individual humans represent “deviations around nature’s ideal,” or both, values around a mean are often considered noise, and the only way to eliminate this noise is to average it away. What we are left with, then, is an approximation to the “true” value for that human dimension. It is this assumption regarding the interpretation of noise, and the truth value of the mean, that we think requires questioning. This assumption has several subsidiary assumptions (see **Table 1**) that we tackle below. In the sections that follow we review each of these assumptions and conclude that they are difficult to justify. So too are some of the implications of using the mean to infer features of the human cognitive system.

**Table 1:** Assumptions underlying the use of the mean in psychology research.

1.There is a true value thatwe are trying to approximate whenwe measure humans on some dimension.
2. Averaging helps us to eliminate the noise in our measures to see the true value.
3. Any inability to use the mean as a reliable measure of a stable characteristic is a product of weaknesses in methodology or calculation (i.e., it does not represent a failure in the initial assumption that a true value exists).
4. The noise in our measurements represents the effects of variables unrelated to the one being measured.

The uncritical or unreflective use of the mean in much psychological research makes us problematically blind to variation and distribution amongst the data we collect. In focusing narrowly on the mean we make ourselves blind to potential variation and complexity in our data and in the cognition and behavior those data represent. The assumptions we identify as underlying the uncritical use of the mean are, effectively, assumptions about certain characteristics of the psychological system, characteristics that tell us more about the theoretical goggles we are wearing than about the behavior we are observing. In later sections of the paper we consider alternative approaches that help us shake these assumptions, and suggest how these may provide fruitful means of conducting research in psychology.

## ASSUMPTION 1: THERE IS A TRUE VALUE THAT WE ARE TRYING TO APPROXIMATE WHEN WE MEASURE HUMANS ON SOME DIMENSION

What is it about human behavior we are trying to eliminate by averaging? In our own field of cognitive psychology it would seem we assume that in each head there is a mechanism that is common to all/most people, but which is obscured by our noisy measures and/or our noisy heads. That is, in our experiments, we expose a group of people to the same conditions. Everyone is assumed to respond similarly to these conditions because their cognitive mechanisms are similar. Unfortunately the data we collect from these people are not identical, and we assume this is because our measurements are not perfect and that there are a myriad of tiny and random effects that conspire to create noise in the data. Still, if we test a sufficiently large sample size, averaging should enable us to observe the characteristics of each cognitive mechanism unobscured by the noise.

The main question that occurs to us when we consider this scenario is why do we assume that everyone has the same cognitive mechanism? Just as we would not readily accept that each person’s height is some deviation from an ideal height, it is odd that we would accept that each person’s brain works in exactly the same manner. Certainly this is the assumption that our research methods in cognitive psychology rest upon, and yet there does not appear to be any attempt to justify it^2^.

One means of justifying this assumption could be to point to other systems in the human body and note that they all tend to work in similar ways in each individual. For example, the heart operates in the same manner in each person and although some viable deviations from the standard exist (e.g., atrial septal defect, dextrocardia), the vast majority of people have similar cardio-vascular systems. Most of the other major systems in the body also have the same uniformity across the human species, from the cellular to musculo-skeletal levels. In response to this justification, however, we would point to the fact that the brain has one major difference to the other systems in the body – it changes its mode of operation as a function of experience^3^ We consider this response further below, but for now we suggest that the assumption of common cognitive mechanisms is one that can be challenged, and probably should not be the starting point in explaining human behavior.

## ASSUMPTION 2: AVERAGING HELPS US TO ELIMINATE THE NOISE IN OUR MEASURES TO SEE THE TRUE VALUE

We have no issue with the common statistical notion of sampling error. This is the notion that, when sampling from a population of scores, each sample will have a mean that is likely to vary from the mean of the population with a fairly predictable probability. That is, there are likely to be many samples with means that fall fairly close to the population mean, and a much smaller number that have means further away from the population mean. The chances of obtaining a sample mean close to the population mean are increased by taking a larger sample.

The problem we have with sampling is more in the interpretation of sampling error. Just as we have a difficulty with the concept of the mean reflecting a true value on some variable, we also find it challenging to accept that sample values on either side of the mean reflect noise in the data. This interpretation suggests that these values are not psychologically meaningful. Instead they are a nuisance factor that requires elimination. Indeed, if this noise did not exist, if we could measure “true” values directly, we would have no need for inferential statistics such as the analysis of variance (ANOVA).

Despite there being something of a tradition within psychology pointing out the difficulty in this assumption (see particularly recent consideration by Doherty et al., 2013, and more classically, Meehl, 1978), standard practice would appear to hold tight to this assumption. Just as there are no obvious justifications for the argument that the mean reflects some true value, we have discovered no explicit attempts to justify the elimination of variance as a “cleaning-up” activity. It just appears to be the done thing.

A crucial mathematical (as opposed to psychological/theoretical) assumption regarding the use of the average to eliminate noise is the shape of the distribution in question. For the assumed Gaussian curve averaging provides us with a clear representation of the center of the distribution, the “noise” to either side being averaged away. In a sobering and landmark paper, Micceri (1989) noted that normal curves are very rare in real psychological data. To calculate the mean in the hope of eliminating noise or getting some glimpse of a “true” or even a typical value hidden in the variation is simply to overlook reality in favor of a comfortingly elegant mathematical ideal.

## ASSUMPTION 3: ANY INABILITY TO USE THE MEAN AS A RELIABLE MEASURE OF A STABLE CHARACTERISTIC IS A PRODUCT OF WEAKNESSES IN METHODOLOGY OR CALCULATION (I.E., IT DOES NOT REPRESENT A FAILURE IN THE INITIAL ASSUMPTION THAT A TRUE VALUE EXISTS)

One of the assumptions underlying averaging is that our methodologies are inherently faulty in that they cannot be expected to provide perfect measures of the variables of interest. To some extent, this assumption is indisputable, considering that even measurements of physical properties (e.g., length, temperature) carry with them conventional measurement error values. In psychology, however, we take this assumption further than in the physical sciences. Although we accept that there are features of the physical environment that will affect the accuracy of any measurements we take, we are also concerned with the validity and the reliability of the measures. Validity reflects whether we are measuring what we think we are measuring. Most often psychological variables are not directly observable so we need to construct measures that are directly observable and argue that these reflect the operation of the unobservable mechanisms we are interested in. Even if we assume that our measures are valid in this sense, the reliability of these measures concerns psychology greatly. Indeed, Psychological Test Theory makes explicit this notion by indicating that each score on a particular test reflects the true value for that person on the test, plus error (Novick, 1966). In cognitive psychology, we do not seem to believe that our measures are capable of producing an accurate reflection of the state of a person’s cognitive system at some point in time. Indeed, if we exposed a person to the same stimuli, under the same conditions, on several occasions, and recorded their reaction times in responding to those stimuli, we would likely average the individual reaction times, on the assumption that each RT could not reflect the “true” RT for that person in that condition.

There is little doubt that there would be variance amongst the RT values recorded in this situation but what is the justification for assuming that each RT is a deviant of the true value? Instead of assuming that each RT is the true value plus some error created by seemingly random processes, could it not be possible that each RT reflects the state of the cognitive system *as it is at that point in time*? By this we mean, behavior in response to the experimental conditions reflects not only the external conditions, but also the state of the cognitive system as the behavior is occurring. The system will be in a different state to the one it was in on the previous occasion when an RT was recorded, and to the state when the next RT is recorded, if for no other reason than the fact that the system has experienced a repetition of the experimental conditions and made the same responses. The assumption then that taking the average of measures from repeated trials will provide a reflection of some stable element of the cognitive system seems fanciful given that the system could not be stable if we keep giving it experiences. This is a psychological reflection of the Heisenberg Uncertainty Principle – by measuring a system, we are influencing the system and hence affecting the very thing we are trying to measure. Unfortunately, averaging several scores will not result in a value that reflects some stable feature of the cognitive system. Instead, this stable feature may not even exist.

## ASSUMPTION 4: THE NOISE IN OUR MEASUREMENTS REPRESENTS THE EFFECTS OF VARIABLES UNRELATED TO THE ONE BEING MEASURED

Part of the justification for averaging scores appears to be the assumption that scores on either side of the mean reflect error. This error can be error of measurement, as considered above, but it can also reflect the operation of many factors that influence behavior. There is recognition in psychology that humans are sensitive to a vast range of variables, and any measurement of one variable is going to show the effects of many of these other variables too (hence the widespread usage in psychology of statistical methods such as Factor Analysis and Structural Equation Modeling). However, we assume that these effects have several characteristics. One is that they are random, and the other is that they operate independently of the variable we are interested in (i.e., the one we are currently measuring). Essentially, then, this error is assumed to be analogous to white noise in a radio signal. As such, calculating the mean is assumed to be just like fine tuning a radio signal – in both situations, noise is eliminated to enable a clearer perception of the signal. Again, though, we wonder what justification there is for such an assumption. Below we consider whether it is appropriate to assume that the variance in a set of scores is a reflection of variables that are random and independent of the variable we have measured.

## INTERPRETIVE CONSEQUENCES OF USING THE MEAN

The mean is often used as if it is a good representation of a group of scores. Clearly it is only used when there is variation amongst scores – if there is no variation, then the scores can be characterized easily as so many scores of the same value. When there is variation amongst the scores, some measure that reflects the middle of the distribution of scores is considered to be a good reflection of the type of score that is observed in that set. As this variation in the set increases, however, the confidence one has in the mean being a good reflection of the group decreases.

This raises an issue with respect to the testing of differences between groups in an experimental design. Standard inferential statistics compare the variation between groups with the variation within groups in order to determine whether the scores in one group are significantly different to those in the other group. In spite of its name, then, ANOVA is rarely used to determine whether differences exist between the variances of two or more samples. Although there is explicit recognition that it is variance we are considering, ultimately the conclusions that are drawn in such situations concern whether the mean of one group is different to the mean of the other group. So, even though the statistical test explicitly considers the degree of overlap between the distributions of scores in the groups, the final conclusion is phrased in terms of whether one mean is significantly larger than the other.

The extent to which such a conclusion is a fair reflection of the state of the distributions is of course affected by the size of the difference between the means but it is also influenced by the amount of variation in the distributions, and the number of scores in each distribution. Thus it is not uncommon to see significant differences between means reported where the differences are very small. This will happen when the variation within groups of scores is small, and/or the number of scores is large (Cumming, 2012, chapter 12).

In the end, though, a conclusion that the mean of one group is different to the mean of another group can end up being translated as one condition improved performance more than another condition, or similar. But is this an accurate summation of the outcome of the statistical test, and for what purposes are such conclusions used? When one concludes that one condition led to better performance than another condition, at best the implicit conclusion is that, on average, or generally, this condition improves performance. But, as is clear from the above characterization of inferential statistics, there may well be significant overlap in the scores between the two conditions. For instance, if Condition A led to better overall performance than Condition B, there could well be scores in Condition B that were better than scores in Condition A. In other words, the final conclusion may represent an accurate description of the state of affairs for a subset of scores, but not necessarily for the whole set. Many undergraduate statistical courses and textbooks include such caveats in the discussion of the outcome of statistical tests, but the practices of scientists and standards of review and publication in journals involve few checks or balances against this kind of concern (see Marmolejo-Ramos and Matsunaga, 2009 for examples and explorations of good practice in this regard).

Inferential statistical tests generally do not provide information regarding the number of cases that do or do not match the pattern of results represented by the difference in means^4^. It is, of course, a trivial matter to generate such information. Doing so can provide illuminating results.

For example, one of us teaches a unit in Cognition in which one laboratory exercise involves replicating the Word Superiority Effect. This is where detection of a letter is found to be more accurate when the letter is presented in the context of a word than when presented in isolation (Reicher, 1969; Wheeler, 1970). Data has been collected in this laboratory exercise for over 5 years. Although the standard word superiority result is found with these data and supported by a statistically significant superiority in the word condition, when individual scores are considered, almost half of the over 500 people in the experiment provided results that either showed no difference between the conditions (i.e., identical accuracy scores in each condition), or showed results that were the opposite of the effect. Although there may well be methodological differences between our experiment and the classic versions published by Reicher and Wheeler, this observation does raise a serious question over the validity of using inferential statistics to assess differences between means. If we just examine the differences between means and focus only on whether or not this difference is statistically significant, we can end up with a conclusion that describes the effect of the manipulation as if it has had the effect on all or most of the individual scores in the data set. In other words, the mean difference can ultimately represent all of the differences, whereas in many situations this may well be inaccurate. To the credit of Reicher and Wheeler, in addition to reporting inferential statistics related to the differences between means, they did also examine the number of people that showed the effect compared to those that did not. Indeed, in their experiments, the proportion was far higher than in ours. The point remains, however, that without investigating the data beyond the means, one’s confidence that the means reflect the overall results should be low (e.g., Balota and Yap, 2011).

One heavy-handed solution to this problem would be to confine our theorizing to situations where the differences between conditions are so clear that there is no need for inferential statistics to determine whether or not differences are significant. An example of such a clear difference between conditions would be where 80% of participants in one condition show results that are higher/larger/better/faster than 80% of participants in another group. This would be a difference in performance that would be obvious, has a good chance of being replicated, and everyone would believe. Confining ourselves to effects that are this obvious would limit the number of phenomena that require explanation, and may reduce the current preponderance of seemingly unrelated phenomena and theories^5^. Although the shortcomings of inferential statistics have been discussed at great length elsewhere, and for some time now (Cohen, 1990, 1994; Hammond, 1996), it would appear that the message is not getting through. Indeed, when we asked some of our colleagues to read early drafts of this paper, a common response was along the lines “everyone knows this stuff.” And yet we see little evidence of a change in behavior. An illustrative survey of the analysis methods used in research reported in 2012 in two prominent cognitive psychology journals is presented in **Table 2**. Perhaps the “everyone knows this stuff” response is a form of the hindsight bias (Hawkins and Hastie, 1990).

**Table 2:** **Number (%) of empirical articles in Memory & Cognition (2012) and Journal of Experimental Psychology: Learning, Memory,and Cognition (2012) classified according to main analysis type**.

Journal	$\overset{-}{X}$/NHST	Ind. Diffs	Other	Total
M&C	88 (82.2%)	17 (15.9%)	2 (1.9%)	107
JEP: LMC	79 (81.44%)	6 (6.19%)	12 (12.37%)	97

*$\overset{-}{X}$/NHST, summary statistics (e.g., mean, frequencies) and null hypothesis significance testing; Ind. Diffs, analysis explored individual differences in responses; Other, analysis could not be classified into one of the other two categories (e.g., structural equation modeling, chi-squared, Bayesian analyses of various kinds, regression or mediational analyses).*

**FIGURE 2:**
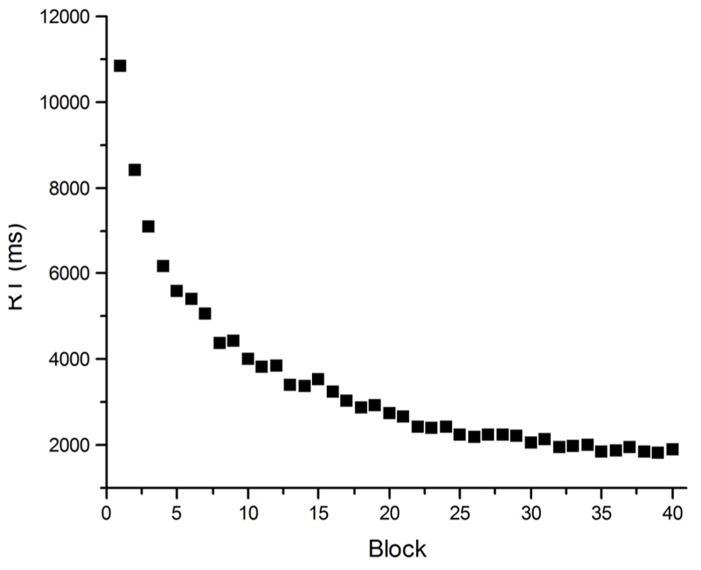
**Mean RT data from practice on an arithmetic task**. Participants (*N* = 40) solved an equation [(*x*^2^ - *y*)/2 = *A*) with eight repeating (*x,y*) pairs, determining whether A was “odd” or “even” for each pair, for 40 blocks of eight trials.

**FIGURE 3:**
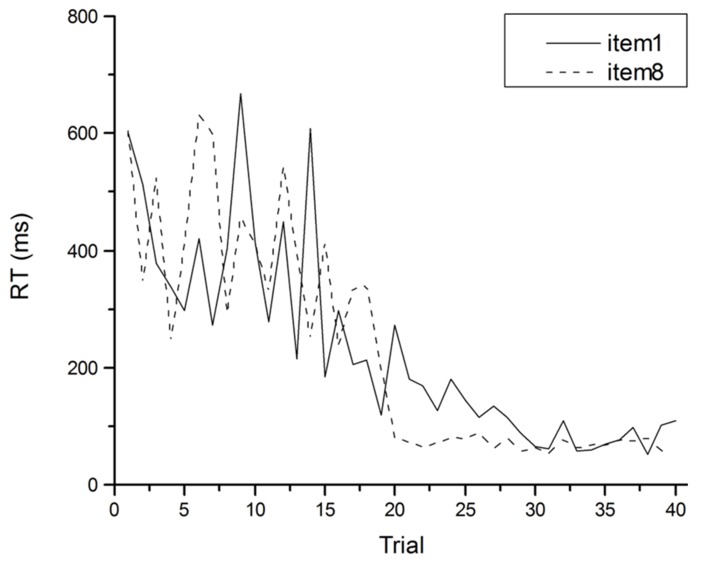
**An individual’s RT as a function of practice for specific items in the arithmetic task of Figure 2**.

An illustration of how problematic averaged data can be comes from Heathcote et al. (2000). The target of their investigation was the Power Law of Learning. This refers to the observation that improvement in the speed of performing a task with practice has a characteristic pattern: performance improves by large amounts early in practice but these increments in performance get smaller as practice proceeds. The smooth trend in these learning curves can more often than not be described well by a power function (see **Figure 2**). Such curves have been observed in fields as disparate as cigar rolling (Crossman, 1959), reading mirror-reversed text (Kolers, 1976) and implicit memory (Kirsner and Speelman, 1996), and are similar to retention and forgetting curves in memory (Ebbinghaus, 1885). So ubiquitous is this observation that it has been said to comprise one of the few laws in psychology (Newell and Rosenbloom, 1981), and the one fact that requires explanation by any credible theory of skill acquisition (Anderson, 1982; Logan, 1988). Heathcote et al. (2000) however, called into question the lawfulness of this relationship between performance speed and practice. They demonstrated that power functions result from averaging any group data with a downward trend. The important point here is that power functions can appear in averaged data, even when they do not occur in individual data. Certainly if individual data is inspected, smooth learning curves are rarely observed. Although performance usually gets faster with practice on a task, performance from trial to trial almost never follows a smooth downward trend (see **Figure 3**).

So, why should theories attempt to explain power function learning if it does not actually exist in individual performance? The assumption of many cognitive theorists appears to be that individual performance does not reflect the real behavior they wish to explain, and that averaging is required over many trials and many people to remove the noise from the data in order for the real pattern to emerge. In the case of the Power Law of Learning, it appears that skill acquisition theorists assume that learning is smooth, and follows a power function, and therefore their theories must posit a learning mechanism that produces power function learning curves. It is possible, though, that this assumption is misguided. Rather than assuming that learning must follow a smooth trajectory, and so average data must be used to observe this smoothness, why not accept that the noise in data is an accurate reflection of the cognitive processes underlying performance? One theory of skill acquisition (Speelman and Kirsner, 2005) does take this position and considers noise in the data as the outcome of competition between cognitive processes striving to control performance. The lesson for all theories of cognitive processes, then, is that proposing mechanisms to explain mean performance may provide explanations of behavior that does not exist.

### ILLUSTRATING THE PROBLEM IN BRAIN IMAGING RESEARCH

Psychology has recently faced a number of controversies that have caused us to take stock of our assumptions and practices as a discipline (e.g., Ritchie et al., 2012 and Roediger, 2012 on replication; Simonsohn, 2012 and Vogel, 2011 on data fraud). Little about the implications of these controversies is really new (Bakan, 1966; Meehl, 1978; Rosenthal, 1979; Cohen, 1994; Kirk, 1996; Rosenthal, 1966) though because of the relatively recent development and excitement of brain imaging research scrutiny in that domain is a particularly burgeoning field (Uttal, 2001; Vul et al., 2009; Carp, 2012), where sources of apparent stability or reliability in a behavior, effect or cognitive process are being increasingly questioned. While there are a number of different statistical and methodological bases for the various concerns raised by critics, the assumption of stability of function and consistency of operation across individuals that averages suggest so strongly is one that has not been carefully considered in much of the localization of function neuroscience literature.

Brain imaging research tends to produce colorful pictures of the brain with specific areas highlighted by bright colors to signify areas of high neural activity, typically associated with cognitive functioning of a particular type. Such pictures, however, are only generated through a process of combining activity patterns across many trials and many people. Data collected from an MRI machine are very noisy. Or at least, that is one interpretation. Another interpretation is that MRI machines produce an incredible amount of data. At any one moment in time the activity of neurons across the whole brain is inferred from the measurement of blood flow. If someone looking at a picture of the activity pattern hopes to see something easily interpretable, it is not surprising that the initial impression is that of a noisy and possibly random assortment of activations of varying degrees. However, if one assumes that hidden amongst the noise are areas of high activation where specific forms of cognitive processing are occurring, then one might consider looking for such areas by trying to eliminate the noise. Much of the noise comes from systematic sources such as the MRI machine and participant movement and so can be easily compensated for. Other noise, however, is seemingly random neural noise. Despite the impression provided by functional magnetic resonance imaging (fMRI) pictures, when areas of the brain are highly active, the other areas of the brain are not quiet. Determining the signal from the noise, then, becomes an important consideration when analyzing the activation patterns. Several methods are used to “clean up” the signal. One involves exposing individuals to many trials involving the same stimuli and requiring the same responses. The activation patterns from similar trials are then combined through averaging. This method assumes that the activation associated with particular stimuli and responses is similar each time. Another clean up method involves combining the average activation patterns from several individuals. One of the problems associated with this averaging step is that there are considerable individual differences in skull proportion. To combine patterns from different heads requires mathematically correcting each skull so that it matches the dimensions of a standard skull. This then ensures that activation patterns are combined from corresponding brain areas.

All of the averaging and correction involved in the analysis of fMRI activation patterns is concerning given our arguments about the mean. In particular we are concerned that fMRI researchers have designed their analytical tools to match their assumptions regarding what they will find in the data. Unfortunately, insufficient critical attention to these assumptions could mean that alternate hypotheses are ignored. For instance, when researchers combine fMRI activation patterns from many people, they assume that brain structures are similar across people, and they are responsible for similar functions in all people. Although we have no argument with the proposition that human brains share the same gross anatomy, we wonder about the assumption that specific localized areas within the cortex are responsible for specific cognitive functions. Some areas of the cortex have undeniable links with certain functions (e.g., the occipital lobe plays a major role in vision), but within certain areas, researchers often try to make the distinctions between an area that could be responsible for cognitive function X, whereas function Y is controlled by a different area (examples abound, see Gläscher et al., 2012 for a recent one). Further, we will get a stronger demonstration of this if we combine the activation patterns of many people, the more the better for the sake of statistical power. The problem with this strategy is that alternative hypotheses – that function is not localized, or that it is, but is localized differently for each person – are ruled out by the methodology. The activation patterns that result from averaging over many trials and many people may not actually reflect what goes on in any one person’s head. It may only be a reflection of what we would see if we undertake a lot of averaging. In other words, the activation pattern may not really exist except as some epiphenomenon of the methodology. This then raises a further problem – how can we make sense of group data when considering the case of an individual. Are we able to generalize a picture that has been derived from many data points to one data point? There are clear practical implications in cases involving brain damage and surgery.

Brain imaging research has over the past decade begun to move away from simple localization research, and significant developments have occurred in areas such as single-trial analyses (for a useful and brief map of that literature see Pernet et al., 2011). Indeed a recent study (Dinstein et al., 2012) that compared brain images from autistic adults and control subjects demonstrates how averaging brain images across trials and individuals can reveal a story quite different to the one that emerges from a focus on individual trial data. Others (Zilles and Amunts, 2013) have explicitly suggested that variability between subjects is not noise but important information. Reviewing neuroimaging work by Mueller et al. (2013) Zilles and Amunts examine a range of ways in which group-based analyses of neuronal structure can lead us to overlook information about individual differences in neuronal structure and change in structure over time that offers crucial clues to the processes underlying brain development. In particular, the use of group means allows different levels of individual variability to affect the sensitivity of methods used to find differences in brain regions (low variable regions will show small effects more noticeably).

While the logic of averaging is clearly problematic in the case of brain imaging research, and is under current active scrutiny, this issue nevertheless remains problematic for other areas of psychology too.

## DOES THE MEAN UNCOVER OR IMPOSE UNIVERSALITY? REFLECTING FINDINGS IN PSYCHOLOGY UPON OUR OWN PRACTICES

The mean, like any piece of technology, is a tool. In itself it is impassive. Any use of a tool, however, is conducted on the basis of standards within a community of practice. While the mean itself is not laden with any particular theoretical assumptions our use of the mean is, and these assumptions are not without consequence.

Our purpose in the present paper is to encourage researchers to more frequently reflect on the fact that in focusing on the mean, in following a tendency to collapse things and encapsulate things into averages, we filter out individual differences and impose universality rather than finding it.

Molden and Dweck (2006) explore some of the ways in which a person’s understanding or interpretation of a situation or phenomenon can have a dramatic effect on their behavior. Whether a person takes intelligence as a fixed capacity (what they term an “entity view”), for instance, or as something that can change or develop over time (an “incremental view”) impacts on how a person performs in learning situations and how they respond to challenges or feedback. It would appear that individual differences in the meaningfulness of the situation can, sometimes dramatically, influence what a person does or what they are capable of, undermining any easy predictions based on what we might understand to be the “typical” cognitive system underlying such performance.

Summarizing several strands of such work, Molden and Dweck (2006) argue that while it is important for psychology to search for universals in behavior and cognition, these universals should be carefully described at the right level of abstraction. Our descriptions of the human psyche, when done in general or universal terms, potentially obscures the ways in which cognition, attitudes, values and behavior vary between people and between contexts.

This potentially limits our science in two important ways^6^. Firstly, by obscuring variation it biases our perceptions of the phenomena we study, making them appear more stable and determinate than may well be the case. Ironically, research in social psychology has warned us of such biases in human perception for decades. It is termed the fundamental attribution error (Jones and Harris, 1967; Ross, 1977), or correspondence bias, and is a notoriously difficult habit to break. Put simply, when we see another person act in a particular way we tend to see the behavior as dispositionally driven, rather than context-dependent. We are more likely to view the behavior as a stable characteristic of the individual rather than as a response to the specific vagaries of the circumstances in which the behavior occurs. To criticize much psychological research as falling prey to the fundamental attribution error would of course be glib and inaccurate but as a notion that is both provocative and evocative it is a useful tool with which to illustrate the problems of overlooking or downplaying variation in people’s behavior or cognitive activity and summarizing outcomes with means alone. We rightly take pride in our use of objective tools in the conduct of our research and analysis but our exuberance for method can lead us to overlook the embedding of these tools in less objective assumptions and standards of practice that need periodic review. It is easy for us to make claims such as “the data show…” when the data can of course be used to support a number of different possible stories, once we have tamed it with data-cleaning techniques and stabilized the outcome with a single summary figure – the average.

The second way in which our science is limited by an over-dependence on the mean as summary is in the generalization of results. The frequent use of the average as the sole description of a group’s performance on a task, or measurement on a trait, characteristic or outcome, greatly limits our understanding of individual cases. It is well known that we cannot predict the individual case from statistics but where our discussions of measurements are presented almost exclusively in terms of averages we constrain ourselves to describing and discussing groups alone. It may be argued, reasonably, that decades of measurements have shown that human beings are so variable in their responses as to make confident predictions of individual’s actions to be foolhardy. There are so many variables, often interacting in non-linear ways, that generalization to the individual simply cannot be a reasonable aim of the discipline.

It is certainly the case that researchers routinely report variability measures (e.g., standard deviations, standard errors, confidence intervals) along with means. Despite this widespread reporting, however, one can question whether researchers are utilizing this information to temper their conclusions that are based upon the means. These measures generally provide information about the size of an underlying distribution but little information about its shape. Given the overwhelming focus upon mean scores, we wonder if the reporting of variability measures is merely an afterthought, or just fulfilling an expectation of journal reviewers and editors. More obviously, the reporting of sample variability measures completely overlooks the variability inherent in individual participants’ responses – information regarding this variability is eliminated by using subject means to reflect each person’s performance.

Nonetheless, examining ranges of scores and variability as phenomena of interest in and of themselves would provide us with a context within which to frame individual observations, the better to understand what the possibilities are such that we can then make a more informed decision about what the *probabilities* might be in the individual case. Rather than seeing outliers as unclean, aberrations or errors that should be excised before the real work begins, they provide us with information on what is possible. Rather than trying to prophecy single specific outcomes, which would likely be unsuccessful, a describing of the landscape of possibilities would provide useful insight in many behavioral contexts.

Context effects are ubiquitous in all areas of psychological research. Our habit of describing things in terms of means rather than ranges and distributions tends to reduce analysis of this fact into a list of independent observations, a shopping list of possibilities with little to relate the differences in cognitive function from one situation to the next. The very term “context” is frustratingly difficult to define, and varies in use from experiment to experiment, researcher to researcher, a lack of discipline that isolates the work of different individuals and thus obscures what relationships exist between the various independent observations, making it difficult if not impossible to overcome the “shopping list” state of our current understanding. (It is left as an exercise for the reader to review what the “context effects” are in their own domain of interest and to examine just what is being considered as “context,” and the criteria on which that decision is based.)

We would argue that the unguarded use of the mean to summarize outcomes from different experiments suppresses the perception of both variability and continuity between results, tempting us to see the differences as more stable and certain than they really are, and leading to the balkanisation of research that limits our insights into psychological functioning. In this, we suggest that the mode of analysis and description inherent in the use of the mean as principal summary statistic is very similar to that of the cognitive linguistic phenomenon of nominalization as described by Barsalou et al. (2010), p. 350,

...it is possible to conceptualize nouns in decontextualized ways, and these decontextualizations play important roles. We err, however, when we mistakenly believe that these decontextualized mechanisms refer to meaningful entities in isolation, and forget that they operate intrinsically in contexts and depend on contexts for their realization. The mechanism indexed by a noun integrates a large system of situated patterns, with this system usually producing an emergent form well-suited to the current situation.

Similarly, the behavior or cognitive activity indexed by a mean of measured performance is a collection of context-sensitive processes that likely include much more than the specific independent variable with which that mean is explicitly associated in a given study. Though it is inherent in psychological training that we be critical and circumspect in our assessment of reported results, we are not immune to the biases that we report in our participants’ behavior.

In no sense do we suggest that the mean is somehow wrong. The problem is rather that it is so satisfying. Decades of research on attribution biases and Barsalou et al.’s (2010) work on nominalization suggests that the kind of encapsulated and stable idea of performance that the mean suggests is an enticing, seductive view (at least for the Western majority involved in high “impact” psychological research). The basic aims of research, insofar as it entails a search for the general and the universal fill our perceptions and interpretations of data and settle standards of practice that lean heavily toward the stable, reliable and consistent. We thus suggest that the problematically uncritical use of the mean is an expression of an unreflectively held view of the psychological system. Re-consideration of our statistical tools will also involve some re-consideration of our theoretical standpoint and the standard ways in which we formulate research questions.

Though there are certainly domains and approaches within psychology that emphasize contextualized performance and situational variability (see for instance Barrett et al., 2010 for a survey of recent cognitive work; see also the much discussed situational view of personality by Mischel and Shoda, 1995; Mischel, 2004), the search for general capacities and universal functions is by far the more common.

## SUPPRESSING THE ASSUMPTION OF STABILITY

There are alternatives to thinking about psychological mechanisms as shared and stable characteristics of the human species that do not lead us into despair or pessimism about the possibility of a unified and systematic theory of psychology. The development, over the past two decades, of modes of thinking that place great emphasis on individual developmental dynamics, the (often messy) details of a cognitive agent’s actual, real, bodily interaction with its environment, provide us with an approach that allows for more nuanced, dynamic perspectives on psychology and psychological processes.

There are a number of these different ways of thinking. They are not necessarily commensurable with one another and as yet do not offer a single coherent vision of psychology that might be recognizable as a “paradigm” in the Kuhnian sense, to which we could leap in some revolutionary fervor. However, what these different approaches make clear, having been developing around the fringes of the discipline for decades and gradually encroaching further into mainstream research, is that valuable, fruitful research can be done in which the emphasis is placed on the dynamics of change in psychological processes over time, and in which the complexity of interactions between the individual characteristics of both the person and the environment in which they are acting can be accounted for and incorporated into scientific psychological theory. Such approaches do not suppress variation in the behaviors of people but rather see it as a rich resource for understanding how psychology interacts with context. Similarly, while these approaches use a variety of statistical tools other than the standard significance testing that remains the mainstream, the arithmetic mean is still used, but its use does not require the assumptions of underlying “true” values clothed in noise with which we take issue in this paper.

The developmental dynamics of Thelen and Smith (1994) are a perfect example of this focus on processes and change over rigid structure. Thelen and Smith examine development as a contextualized process of interplay between the child and their environment, providing evidence for the growth of motor skills not as the blossoming of standard, universal cognitive capacities (true values to be approximated with averaged observations) but as the coping of the individual child with the demands of their idiosyncratic histories. The differences between children in their development has at least as much to tell us about how development occurs than the similarities.

The idea that the cognitive system is not rigidly specified, but is in fact supple and responsive (over a number of timescales) to the quirks, specifics and details of the environment in which it operates is summed up byClark’s (1997) description of the “soft assembly” of cognitive function. Rigidly or “hard assembled” systems have a fixed structure and mode of operation. There is a “right” way to describe how the different components of the system relate to one another, an ideal of the system that is, in some fundamental way, correct. Not so for soft assembled systems.

Soft assembled systems tend to have loosely inter-connected components, less fixed positions within a structure so much as a pool of potential resources that can be organized within various constraints in response to situations and task demands. There is no ideal of how such a system should be organized, no schematic that can be drawn that captures the correct way in which the components might relate to one another, as these things will vary continuously depending on contexts, individual histories and immediate requirements. Soft assembled systems tend not to use central controllers but rather they self-organize, with task-specific activity emerging from the dynamic interaction between components and environment. This can happen either over quite brief timescales, or more slowly over longer periods.

The net result, if cognitive activity is assembled in such a manner, is that similar behavior might in fact be the result of quite differently organized psychological processes. There is no “correct” mapping of the psychological system, no signal about cognitive structure being hidden by the noise of individual variation. Such a theoretical standpoint eschews assumptions of single true values to be sought in the noise of individual variation and measurement error. What must be understood is the dynamics of response to situations over time, with an appreciation that different individual histories will often result in quite differently arranged but similarly performing psychological systems. What is more, it may be the case that even within a single individual over particular timescales (those associated with learning in its many forms) we might see the structure and functioning of the cognitive system changing dramatically.

## UNDERSTANDING SIMILARITY AND STABILITY IN BEHAVIORAL PERFORMANCE

The idea of a stable and shared set of basic cognitive processes underlying some of our use of the mean is not entirely an assumption. It is rooted in the success of our everyday interactions, the ease with which we can coordinate with one another, share experiences and activities. A critical reader would no doubt at this point be arguing that the statistical tests we typically use in data analysis invariably take the variance or deviations within the sample into account, while also emphasizing the plain fact that while it might be true that everyone is unique, it is plainly true that we share a great deal. People vary, sure, but looking around, they do not vary nearly as much as they could in most cases, and most of what differences do exist seem to be quite subtle – certainly nothing requiring any fundamental re-think of our use of statistics or theoretical perspective.

Amongst these new approaches to psychological research, how is this simple truth about the similarity of human beings to be captured and explained?

There are certainly some things that human beings generally share (though there are very few that are genuinely universal). Our basic body plan, our nutritional requirements, the range of physical stimuli to which we are sensitive and with which we can interact, these tend to vary within fairly narrow ranges. These shared constraints on our behavior will provide particular channels for developmental change, channels that will be structured further by the cultural provision of particular developmental tasks and demands. Each human being follows a unique developmental trajectory but there are constraints on that trajectory. A key observation here, however, is that this more developmental mode of explanation suggests that the reason for people’s similarities is not the inexorable unfolding of a pre-specified and consistently presenting cognitive system, but the shared constraints on development, which may specify the ends (consistency in behavior), but will typically underspecify the means.

The developmental work of Thelen and Smith (1994) once again offers us some examples. Their much cited work examining the development of reaching and grasping in two infants, Hannah and Gabriel, describes how features of the children’s bodies, their intrinsic dynamics, idiosyncrasies of energetics or even simply mass, mean that each child has a different developmental task in order to achieve the same outcome. Whereas the excitable Gabriel must learn to draw energy out of the whole reaching system and slow his movements down if he is to manage to get his hand successfully to a target object, the more placid Hannah must learn to put more oomph and effort in to achieve the same result. Karmiloff-Smith (2000) describes similar differences, this time not in order to explain individual differences but rather to explain the response to different developmental constraints in the performance of the “standard” function of face recognition for people with or without Williams syndrome (WS). She argues that similarly proficient behavior of people with or without WS in the overall task of face recognition is underpinned by quite variant collections of more basic cognitive skills (e.g., recognizing facial identity, facial emotion, eye-gaze direction, or lip-reading). That is, the same behavioral outcome can be the result of quite different forms of underlying process. This is a clear case where one researcher’s noise is another researcher’s signal.

Typically as researchers it is precisely those choices and manipulations of the environment that will ensure the highest probability of similar performance are those most highly valued in experimental settings. In most of our research we take great pains to limit the range of participants’ behaviors in order to make consistency and similarity the most likely outcome. Gross variability, or indeed any variability not directly attributable to the chosen independent variable is considered a sign of a poorly designed study – other potential sources of change and difference are suppressed. There are certainly times when this is desirable. We suggest, however, that such practices have been adopted as standard and implemented by many researchers without due consideration of their valid range of application. Recently developing theoretical perspectives within the dynamical family of viewpoints push explicit reflection on these questions to the fore once again. We consider this a very positive development, one which will not suppress the use of the mean in our research, but will hopefully suppress its use in an uncritical, or overly focused manner.

## CONCLUSION

Our point in raising these issues is not to suggest that psychology wean itself off use of the mean, or to go cold turkey on averages. Such inane recommendations would deserve the disdain with which they would inevitably be met. However, we do argue for a more careful, critical and explicit use of averages in the discussion of measurements and the reporting of results. Specifically, we argue that the mean must not be used without reflection on the theoretical assumptions and frameworks that are underlying its use and we suggest that in the typical case a theoretical perspective closer to that of dynamical systems will be more appropriate, providing more context and a fuller picture of the behavior in question from the data observed.

The average provides us with important and useful information but we see its use in summarizing and analyzing groups to suppress important individual differences in behavioral and cognitive performance as having become unbalanced. The range and variance of scores in distributions should be reported as frequently and clearly as averages and should temper our easy acceptance of the mean as representative of the numerous individual people whose behavior or characteristics are being recorded. Tukey (1977) pioneered graphical techniques for presenting such information. An excellent recent example is provided by Doherty et al. (2013), whose presents the means from a one-way ANOVA design, along with all of the data that featured in the analysis. This figure not only depicts the relationship between the independent variable and means on the dependent variable, but it also reveals the extent to which the relationship exists amongst the individual observations, and represents the overlap between conditions more completely than a group of error bars or confidence intervals. Other examples already mentioned are those of Marmolejo-Ramos and Matsunaga’s (2009) work on graphical techniques in exploratory data analysis and Balota and Yap’s (2011) suggestions about “moving beyond the mean” in analysis of reaction time curves. Readers are also pointed toward Landau’s (2002) introduction to survival curves, which allow for the mapping of relationships between variables and outcomes over time in a simple but clear manner.

Rather than focusing purely on the question of whether a difference exists, our aim should be to use statistics to illustrate and characterize the range of measurements recorded as fully as possible. By using the range of quantitative options available more fully (range, median, variance, and others) we can provide a better qualitative appreciation of the behaviors we observe, a richer and more nuanced picture of the phenomena that we are interested in describing, explaining and predicting. This will also allow our predictions to become much more interesting – not just whether one group will be bigger or faster or more but what the range or distribution of outcomes are likely to be depending on the size of the sample or its composition. Further, we could examine whether there are differences on a range of variables (e.g., working memory capacity, IQ, reading speed) between people who do and do not show the average target effect. This will provide us with a richer data source that may reveal more about why some people exhibit the effect and others do not, and this would expose more information about the mechanism under scrutiny.

We would also do well to expand the set of tools available to us. The history of psychology is rich in alternate methods of analyzing behavior that do not rely on averaging group data. Research in psychophysics regularly analyses data from individual subjects; neuropsychology has a long history of single case studies; Piaget’s theories were developed on the basis of analyses of the behavior of a few subjects. In cognitive psychology, however, although there are many researchers who fit mathematical models to individual data (Lewandowsky and Farrell, 2011), the modal behavior is to focus on grouped data and average performance (see **Table 2**). Lewandowsky and Farrell, 2011 (p. 106) suggest

that it may be advisable to fit one’s models to both individual data and aggregate data; if both yield the same result, one can have increased confidence in the outcome. If they diverge, one ought to rely on the fit to individuals rather than the aggregate.

With such data, dynamical and complex systems thinking offers rich possibilities for alternate modes of investigation, as does Bayesian analysis. Of course, these new instruments would not exempt us from our role as sensitive, judicious and critical tool users any more than would our more widely practiced and familiar analytical techniques.

The mean’s many roles should be clear in our minds as we design and conduct our experiments, as we take measurements, and carry out analyses. Interpretations of the results should be limited accordingly. Such critical consideration of the mean may prompt us to broaden our methodological horizons, balancing a sensitivity to the potentially universal and broadly shared with the unique, the variable and the idiosyncratic. Ultimately, we should be mindful of the purposes for which we are using the mean and more importantly, the things we are trying to reflect by using the mean. We should consider the degree to which we can assume that the people from whom we take measurements and calculate average scores all possess a similar cognitive mechanism that underlies the performance we have measured. If we think there is likely to be a high degree of similarity in mechanisms, then reflecting that performance with a mean is justified. Otherwise, the mean will severely obscure variations in performance and hence the variety of cognitive mechanisms possessed by people. Finally, as researchers, we should deliberate over whether we are making the assumptions about the mean that have been highlighted here. If these assumptions are implicit in our methodology, then we should consider whether each is justifiable in our particular research context. If they are not justifiable, then alternate tools may be necessary.

## Conflict of Interest Statement

The authors declare that the research was conducted in the absence of any commercial or financial relationships that could be construed as a potential conflict of interest.

## Footnotes

1 Other distributions are also observed with respect to measurements of human characteristics, but these often reflect something peculiar to the characteristic under scrutiny. For example, log-normal distributions are predominant in situations involving some degree of competition or interaction between the elements being measured (e.g., Halloy, 1998). Reaction time distributions (seen as ex-Gaussian distributions) are invariably positively skewed, and this has inspired much model development and testing (e.g., Ratcliff and McKoon, 2008; Holden etal., 2009; Heathcote and Love, 2012). Other distributions are also observed with respect to measurements of human characteristics, but these often reflect something peculiar to the characteristic under scrutiny. For example, log-normal distributions are predominant in situations involving some degree of competition or interaction between the elements being measured (e.g., Halloy, 1998). Reaction time distributions (seen as ex-Gaussian distributions) are invariably positively skewed, and this has inspired much model development and testing (e.g., Ratcliff and McKoon, 2008; Holden etal., 2009; Heathcote and Love, 2012).

2 This is the strong version of the assumption. The weak version is that we assume that people have *pretty much the same* cognitive mechanism. This, however, is not a version that helps. If we accept that people have slightly varying cognitive mechanisms, using the mean to draw inferences about these mechanisms – that is, to get a picture of some average mechanism – we end up with a mechanism that may not exist in anyone’s head. More on this later.">This is the strong version of the assumption. The weak version is that we assume that people have *pretty much the same* cognitive mechanism. This, however, is not a version that helps. If we accept that people have slightly varying cognitive mechanisms, using the mean to draw inferences about these mechanisms – that is, to get a picture of some average mechanism – we end up with a mechanism that may not exist in anyone’s head. More on this later.

3 Other systems in the body do this also (e.g., the cardio-vascular system responds to regular exercise by becoming more efficient), but this is usually in a quantitative manner, whereas the brain alters its functioning in not only a quantitative, but also a qualitative manner (Patel etal., 2013). Other systems in the body do this also (e.g., the cardio-vascular system responds to regular exercise by becoming more efficient), but this is usually in a quantitative manner, whereas the brain alters its functioning in not only a quantitative, but also a qualitative manner (Patel etal., 2013).

4 See Cohen (1977) for a measure (U3) that estimates such group differences *post hoc*. See Cohen (1977) for a measure (U3) that estimates such group differences *post hoc*.

5 Other solutions have been proposed before. One is to report effect sizes along with the results of significance testing (Cohen, 1994). This does not, however, address the issues we have identified because effect sizes are mostly used in comparisons of means. In this context the effect size is just a way of characterizing how large the difference is between the means, in relation to the variance observed. Another solution is to report confidence intervals with means (Cumming and Finch, 2005). Again this does not entirely solve the problem because a confidence interval is just a measure of average variance, and so glosses over details of a distribution of scores. Balota and Yap (2011) demonstrate that several parameters describing RT distributions can be psychologically meaningful.">Other solutions have been proposed before. One is to report effect sizes along with the results of significance testing (Cohen, 1994). This does not, however, address the issues we have identified because effect sizes are mostly used in comparisons of means. In this context the effect size is just a way of characterizing how large the difference is between the means, in relation to the variance observed. Another solution is to report confidence intervals with means (Cumming and Finch, 2005). Again this does not entirely solve the problem because a confidence interval is just a measure of average variance, and so glosses over details of a distribution of scores. Balota and Yap (2011) demonstrate that several parameters describing RT distributions can be psychologically meaningful.

6 Molden and Dweck and other’s research noted here uses mean group differences in precisely the way we are criticizing. The point is not that such research is not to be done, or is somehow valueless (far from it) but it must be interpreted and used judiciously, and we should look to what is possible given such conclusions rather than what is somehow essential. In the present case, we should be conservative about our use of certain statistical tools precisely because it is quite *possible* that it will negatively affect our science, as discussed further in the text.">Molden and Dweck and other’s research noted here uses mean group differences in precisely the way we are criticizing. The point is not that such research is not to be done, or is somehow valueless (far from it) but it must be interpreted and used judiciously, and we should look to what is possible given such conclusions rather than what is somehow essential. In the present case, we should be conservative about our use of certain statistical tools precisely because it is quite *possible* that it will negatively affect our science, as discussed further in the text.
